# Characterization of an outbreak of malaria in a non-endemic zone on the coastal region of Ecuador

**DOI:** 10.7705/biomedica.5816

**Published:** 2021-05-31

**Authors:** Diego Omar Morales, Paul Andrés Quinatoa, Jaen Carlos Cagua

**Affiliations:** 1 Centro de Referenda Nac¡onal de Vectores, Instituto Nacional de Investigac¡ón en Salud Pública, Quito, Ecuador Instituto Nacional de Investigac¡ón en Salud Pública Quito Ecuador; 2 D¡recc¡ón Nac¡onal de Prevenc¡ón y Control, Ministerio de Salud Públ¡ca, Quito, Ecuador Ministerio de Salud Públ¡ca Quito Ecuador

**Keywords:** Malaria/epidemiology, Plasmodium vivax, d¡sease outbreak, trans¡ents and m¡grants, malaria/epidemiología, Plasmodium vivax, brotes de enfermedades, m¡grantes, reporte

## Abstract

**Introduction:**

Malar¡a ¡s a vector-borne d¡sease w¡dely d¡str¡buted ¡n the Amazon reg¡on and the coastal area of northern Ecuador. Its ep¡dem¡ology ¡nvolves related factors such as human settlements, vector reproduct¡on s¡tes, mob¡l¡ty, product¡ve act¡v¡ty, and the response capac¡ty of health systems, among others.

**Objective:**

To describe malaria transm¡ss¡on by *Plasmodium vivax* ¡n a non-endem¡c area of Ecuador by analyz¡ng the ep¡dem¡olog¡cal and entomolog¡cal factors ¡nvolved.

**Materials and methods:**

We conducted the epidemiological study of the cases reported ¡n the Sal¡nas canton and the character¡zat¡on of vector breed¡ng s¡tes through captures of larvae and adult mosqu¡toes by human capture of rest¡ng mosqu¡toes.

**Results:**

We detected 21 cases of malar¡a w¡th local transm¡ss¡on related to the presence of ¡n¡t¡al cases ¡n Venezuelan rrrigrant pat¡ents and ¡dentified *Anopheles albimanus* as the predom¡nant vector ¡n natural breed¡ng s¡tes such as estuar¡es, wells, and water channels.

**Conclusions:**

We detected an outbreak of malar¡a tr¡ggered by ¡mported cases from Venezuela. Cl¡mat¡c, soc¡al, env¡ronmental, and ecolog¡cal cond¡t¡ons have favored the development of the vector maintaining the transm¡ss¡on cycle. Strateg¡es to control ¡mported malar¡a should be mult¡ple ¡nclud¡ng early case detect¡on and control of product¡ve breed¡ng s¡tes to avo¡d local transm¡ss¡on.

## Resumen

**Introducción.:**

La malar¡a o palud¡smo es una enfermedad transm¡t¡da por vectores, ampl¡amente d¡str¡bu¡da en la reg¡ón amazón¡ca y en la zona costera del norte del Ecuador. Su ep¡dem¡ología ¡nvolucra factores relac¡onados, como asentam¡entos humanos, s¡t¡os de reproducc¡ón del vector, mov¡l¡dad, act¡v¡dad product¡va y capac¡dad de respuesta de los s¡stemas de salud, entre otros.

**Objetivo.:**

Descr¡b¡r la transm¡s¡ón de malar¡a por *Plasmodium vivax* en un área no endém¡ca de Ecuador, med¡ante el anál¡s¡s de los factores epidemiológicos y entomológ¡cos ¡nvolucrados.

**Materiales y métodos.:**

Se h¡zo el estud¡o ep¡dem¡ológ¡co de los casos reportados en el cantón de Sal¡nas y la caracter¡zac¡ón de cr¡aderos del vector con capturas de larvas y adultos med¡ante la captura de mosqu¡tos en reposo.

**Resultados.:**

Se detectaron 21 casos de malar¡a con transm¡s¡ón local relac¡onados con la presenc¡a de casos iniciales ¡mportados de Venezuela. Se ¡dent¡ficó *Anopheles albimanus* como el vector predom¡nante en cr¡aderos naturales como estuar¡os, pozos y canales de agua.

**Conclusiones.:**

Se detectó un brote de malar¡a desencadenado por casos ¡mportados de Venezuela. Las cond¡c¡ones cl¡mát¡cas, soc¡ales, amb¡entales y ecológ¡cas han favorec¡do el desarrollo del vector, manten¡endo el c¡clo de transm¡s¡ón. Las estrateg¡as para controlar la malar¡a ¡mportada deben ser mult¡facét¡cas, e ¡nclu¡r la detecc¡ón temprana de casos y el control de cr¡aderos product¡vos para ev¡tar la transm¡s¡ón local.

Malaria is a vector-borne disease widely distributed in tropical and subtropical areas, which is endemic in 97 countries. It is caused by protozoan parasites of the genus *Plasmodium* transmitted by *Anopheles* mosquitoes. Among the 465 species in the genus, only 70 have been reported as vectors of *Plasmodium malariae*, *P. ovale*, *P. falciparum, P. vivax,* and *P. knowlesi* to humans ^1^^,^^2^. According to the World Health Organization (WHO), about 228 million cases of malaria were reported worldwide in 2018 with 405,000 deaths mainly in Africa and in the South-East Asia Region ^3^.

In the American continent, the disease continues to be an important public health problem as there are 102 million people at risk of infection and at least 28 million living in high-risk locations (>10 cases/1.000 inhabitants) ^4^. The Amazon basin area (Brazil, Perú, Ecuador, Venezuela, and Colombia) hosts 44 % of the malaria cases reported in the Americas ^5^. 

Malaria in Ecuador has been one of the leading public health concerns. In 2001, 106.641 cases were reported while in 2014, a 98% (242 cases) reduction had been achieved with the financial support of the Amazon Network for the Surveillance of Antimalarial Drug Resistance (RAVREDA) project *Control de la malaria en las zonas fronterizas de la región andina: un enfoque comunitario* (“Malaria control in border areas of the Andean region: A community approach”) (PAMAFRO). Thanks to this remarkable achievement, Ecuador was included in a group of 21 candidate countries in the world to eliminate this disease by 2020 ^6^. However, since 2014, there has been a continuous increase of cases: 686 in 2015 and 2,081 in 2019 (with no increase of mortality attributable to malaria), which may be explained by the critical strategic changes introduced in epidemiological surveillance and vector control programs ^7^.

Malaria transmission zones in the country are mainly concentrated in the Amazonian provinces bordering Perú and the northwestern coastal zone bordering Colombia. During 2019, 790 cases were registered in provinces of the Amazon region: Morona Santiago with 521 cases in Pastaza and 372 in Orellana, i.e., 84% of active transmission in the country ^7^. In these areas, malaria transmission is related to rainfall increases favoring the availability of habitats for larvae, the development of settlements in forest areas, oil or mining extraction activities, and the migration from neighboring communities in Colombia or Perú, with the subsequent risk of introducing parasites in decreased-transmission areas or maintaining transmission in active areas ^8^.

In the Ecuadorian coastal region, the province of Esmeraldas is the only historical area with cases of *P. falciparum* malaria with 146 cases in 2019 ^7^. There, the presence of asymptomatic cases not detectable by microscopic examination and the instability of malaria control activities may have resulted in the underestimation of malaria cases ^9^. Santa Elena, located in the southern coastal region of Ecuador, recorded only 13 *P. vivax* malaria cases from 2008 to 2013 and since then, it has been considered free of malaria, although its environmental determinants are ideal for mosquito breeding sites and there are socioeconomic risk conditions that intervene in the transmission dynamics. However, during 2019 and the beginning of 2020, 21 new cases were registered, which alerted the epidemiological surveillance and control programs ^10^^,^^11^.

Malaria transmission in a non-endemic area is an unusual event, but it is possible under certain circumstances. These outbreaks are mostly triggered by parasites carried by travelers who return to their country or migrants who come from endemic areas or, less often, by parasites in blood or fluid transfusions unleashing nosocomial infections ^12^^,^^13^.

Malaria epidemiology is very complex because it involves determinants such as the access to health facilities, the type of housing, the proximity of human settlements to vector breeding sites, the abundance of vectors, the socioeconomic status, the mobility, the productive activities, the presence of domestic animals, and the response capacity of health systems. Therefore, it is necessary to understand the link between malaria transmission, climatic variables, and other human-related factors to develop appropriate measures to significantly reduce transmission and achieve elimination in non-endemic areas ^14^.

In the present study, we described the presence of *P. vivax* infection among the population of a non-endemic area for malaria transmission in the city of Salinas (Santa Elena province). We analyzed transmission drivers such as the current social situation and the possible routes of vector transmission underlining the need to maintain an operative malaria surveillance system to prevent future outbreaks.

## Materials and methods

### Study area

Salinas (Santa Elena province) is located on Ecuador's coast (02° 13 'S, 080° 58' W) at sea level. Its temperature ranges from 26 to 28.5°C at 07:30 to 31 to 36°C at 12:00, and 31.5 to 34°C at 16:00. There is a single rainy season (from January to April) with values below 500 mm per year ^15^.

Salinas' economy centers on trade, fishing, and oil-related activities and it is one of the most popular tourist destinations in the country, although the majority of its native population is dedicated to fishing. There are clear social differences with predominantly marginal urban sectors with no access to sewage. Instead, there are rainwater channels on the soil where water stagnates when not properly maintained or cleaned turning them into mosquito-breeding sites.

The city has health centers and health care teams, as well as a general hospital and two basic hospitals, one of them with an accredited microscopist for malaria diagnosis. As Salinas is a small city, access to malaria diagnosis from the furthest point of the city does not exceed 60 minutes. However, as there were no malaria case reports for several years, treatment was not available in the city's health centers. Community participation in health activities is carried out in health centers through the local citizen health committees, but malaria had not been on the agenda of their plenary sessions ^15^.

### Epidemiological study

The epidemiological study for reactive case detection was conducted for the first febrile and afebrile cases detected from April, 2019, through to February, 2020. The scope of active case-detection was 3 km around the homes of the initial cases and involved six neighborhoods. Detection teams included health care, promotion, and vector control teams, as well as neighborhood authorities. For passive case detection in health centers, a microscopic examination (thick blood smear) was performed on all patients with fever while rapid diagnostic tests (RDT, SD BIOLINE Malaria Ag) were used for reactive case detection. Positive cases using rapid diagnostic tests were confirmed by a microscopic examination. Both tests fulfilled the requirements of the quality assurance system.

Finally, epidemiologists had exchanges with district authorities and medical personnel to gain a broader understanding of the social determinants of malaria transmission affecting the areas of study and the possible hypothesis of transmission. Case follow-up and control measures were some of the aspects discussed. With this information, a descriptive analysis based on time, place, and population was implemented.

All the activities carried out as part of the outbreak control and strengthening of the surveillance system were based on the World Health Organization's Reference Manual for Malaria Surveillance, Monitoring, and Evaluation ^16^.

### Entomological study

**Figure 1:**
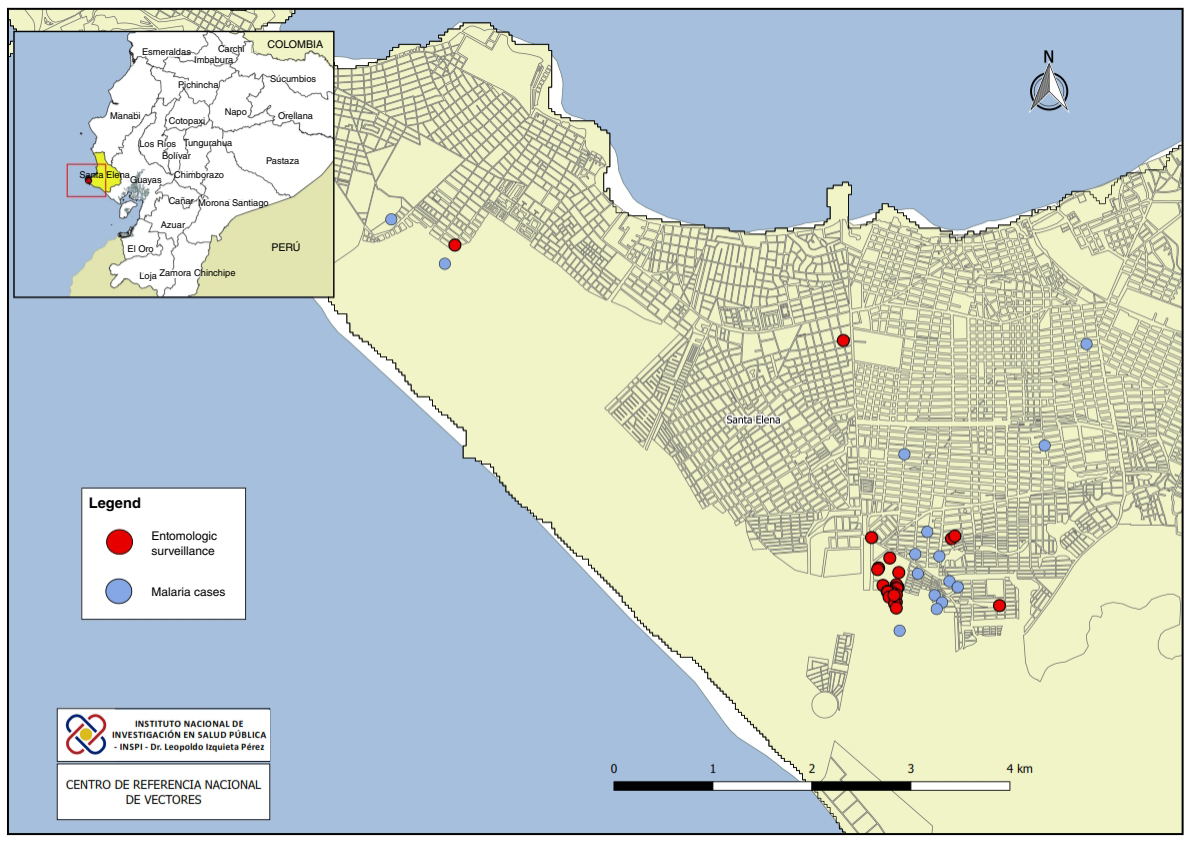
Entomological research locations for malaria, Salinas-Santa Elena province

Entomological samples were collected by the National Vector Reference Center in houses with malaria cases and in the area around them to characterize the breeding sites and determine the abundance of the vector. Immature stages of mosquitoes were collected using plastic pipettes in domestic containers and dippers in natural breeding sites (puddles, wells, channels) (figure 1).

Adult mosquitoes were collected using CDC light traps and a Prokopack motor vacuum aspirator (model 1419). CDC light traps were placed outside four of the homes of confirmed malaria cases from 6:00 p.m. to 7:00 a.m. for three nights, and the Prokopack aspirator was used to capture resting mosquitoes from 18:00 to 21:00 in two houses for two nights. The sampling was done by two persons who took turns every hour to balance possible biases in capture capacity. The samples collected from immature stages were stored in plastic containers. The adult mosquitoes collected with the CDC light traps and the Prokopack aspirator were individually stored in 1.5 ml Eppendorf tubes.

Each sample was identified by date, collection method, and location and then transported to the entomology laboratory of the National Vector Reference Center. For the taxonomic identification of mature mosquito stages, we used the Faran & Linthicum (1981) and González & Carrejo (2009) keys. The samples were identified using a stereoscopic microscope (Carl Zeiss, Germany, model V8 10X / 23 mm).

The epidemiological data and the information on vector control activities came from the early warning system SIVE Alert of the Ministry of Health of Ecuador and the Salinas Health District.

### Statistical analysis

We used ANOVA and t Student tests to compare case numbers using the SPSS™, software package, version 21.0. The level of significance was established at 5%.

## Results

### Epidemiological study

The first malaria case was reported on April 12, 2019, in a Venezuelan citizen whose symptoms first developed on April 8 and was diagnosed with malaria on April 12 at Hospital La Libertad. The patient had had previous episodes of the disease in his native country but did not complete the therapeutic scheme and did not know which it was. The patient had arrived in the city two months before his symptoms began. *P. vivax* malaria was diagnosed, but the hospital did not have the medications, which arrived two days after the diagnosis; however, the treatment could not be given because the patient left the city. The case was classified as imported, probably due to a relapse.

Three months after this initial case, on July 26, 2019, another case was reported again in a Venezuelan citizen whose symptoms started on July 21 and was diagnosed on July 26 by microscopic examination; the parasite was identified as *P. vivax.* That same day, she started treatment with a regimen of chloroquine and primaquine. She said she had no relationship with the initial case, and her address was 10 km away from that first case. The case was classified as imported, although she reported not having been diagnosed with malaria in her country.

After several months of absence of cases, on November 14, 2019, two more cases were reported, this time in Ecuadorians whose homes were a few meters away from the second imported case. *Plasmodium vivax* was identified by microscopy as the parasitic species. These cases were classified as autochthonous and the patients received the same scheme with chloroquine and primaquine.

The notification of these two cases of autochthonous transmission triggered the reactive search within a radius of 3 km involving six neighborhoods. We examined 167 people and detected three cases of *P. vivax* malaria after which, the passive case detection was reinforced in all the city's health establishments and diagnostic tests were carried out on all febrile cases using diagnostic tests, with a total of 189 people examined and one case confirmed by microscopic diagnosis.

**Figure 2:**
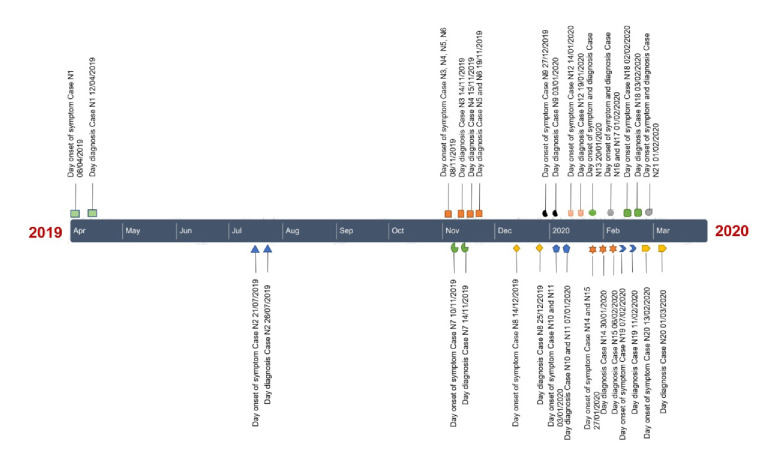
Timeline of malaria transmission in Salinas

A second round of reactive search was scheduled 14 days later, on December 23, 2019, during which we detected a case of malaria among the 15 people examined. In the passive surveillance of cases carried out by the health centers, a case of malaria was reported in a neighborhood far from the initial cases and, thus, the active search for cases in four more neighborhoods was reactivated and nine *P. vivax* malaria cases were detected. In February 2020, another case of malaria was reported in a health center close to the evaluated locations triggering the reactive search for cases. We found two *P. vivax* malaria cases among the 62 people examined. No more cases were detected by passive search and the reactive search was stopped (figure 2).

All cases were hospitalized to receive treatment with chloroquine (10 mg/ kg on day 1, 7.5 mg/kg on day 2, and 7.5 mg/kg on day 3) and primaquine (0.50 mg/kg for 7 days) to ensure therapeutic compliance. After discharge, the closest health center followed up the cases weekly for two months. The majority of the cases in Salinas were men (52.4%) with a statistically significant difference associated with sex (p=0.030). The 20 to 49 (47.62%) age group registered the highest number of cases, but there was no significant difference among the age groups (p<0.007). The predominant occupations of the patients were studying and unemployment (table 1). The transmission most likely occurred in the households and surroundings, as the population was concentrated. The first cases in this location were registered in migrants from an endemic country (2/21; 9.52% of the total cases), and transmission was re-established in a receptive locality (19/21; 90.48%) that had been an endemic area for malaria transmission in previous years.

**Table 1:** Sociodemographic and epidemiological characteristics of the cases reported in Salinas, Santa Elena

Variable	Sample (N=21)	
	n	%
Gender		
Man	9	42.86
Woman	12	57.14
Age group (years)		
0 a 4	3	14.29
10 a 14	3	14.29
15 a 19	4	19.05
20 a 49	10	47.62
50 a 64	1	4.76
Nationality		
Ecuadorian	19	90.48
Venezuelan	2	9.52
Place of residence		
La Libertad	20	90.48
Salinas	1	9.52
Occupation		
Housewife	4	19.0
Merchant	1	4.8
Student	7	33.3
Seller	1	4.8
Administrator	1	4.8
Unemployed	7	33.3

### Entomological study

We identified 203 mosquito larvae. *Anopheles albimanus* was predominant (73%; n=149) in the breeding sites. Other species such as *Aedes aegypti* and *Culex* spp. were identified sharing breeding sites such as tires and plastic tanks with *An. albimanus.*

We found 12 breeding sites with *An. albimanus* larvae. Vector abundance in estuaries was 30.8% (n=46), in wells, 28.8% (n=43), in water channels, 20% (n=30), in plastic containers, 10% (n=15), and in tires, 4.69% (n=7), while in the other breeding sites with puddles the abundance was less than 2.68%.

There were many algae and aquatic plants in the breeding sites at estuaries with a continuous flow of residual discharges crossing Salinas canton and emptying into the Pacific Ocean. During the winter season, increased water flows form small puddles that can be potential breeding sites for the vector. Moreover, the water channels contained residual water from agricultural and domestic activities and they are usually covered with abundant vegetation.

Another main breeding site for mosquitos are wells, usually placed near the houses, used to serve as a source of water before the installation of the potable water service in the locality; now we observe that these wells are abandoned and during winter they collect rainwater and become potential breeding sites for the malaria vectors. We did not analyze the physicochemical properties of the waterbodies, it is assumed that *An. albimanus* was established in the locality given the favorable conditions for its development and their competence in malaria transmission by *P. falciparum* and *P. vivax* along the coastal region.

After the first patient was admitted, *An. albimanus* could have ingested infective gametocytes and started the transmission cycle locally.

We implemented integrated vector management activities for the control of the malaria outbreak in the locality including indoor residual spraying and the delivery of insecticide-treated nets. In a radius of 3 km, a total of 112 homes were sprayed and 132 insecticide-treated nets were delivered, i.e., 84.85% (112/132) coverage for spraying and 64.32% (132/189) for mosquito nets. At the time of the intervention, the lack of insecticide-treated nets prevented a higher coverage. Additionally, with the support of local citizen health committees, we gave talks on education and communication strategies to improve awareness about the disease in the population. Nowadays, there are abundant natural breeding sites for vector development in the locality and vector control measures have not been successful for larval hatcheries, so we focus more on the control of adult mosquitoes. However, there are abundant natural breeding sites for the development of the vector in the locality.

**Figure 3:**
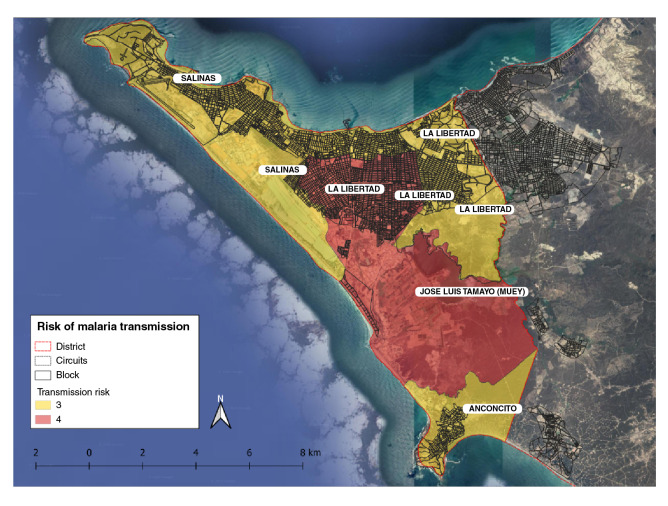
Stratification of risk level for malaria transmission in Salinas, Santa Elena province

The data collected from the epidemiological study was stratified based on receptivity and vulnerability (risk of parasite importation) to transmission. Receptivity was characterized according to the environmental conditions, altitude, temperature, and permanent residency while the vulnerability was evaluated based on the analysis of case occurrence in the previous 10 years and the mobility from/to endemic localities. At the national level, malaria transmission risk is classified into four strata: Stratum one comprises non-receptive areas; stratum two includes receptive but not vulnerable areas; stratum three, receptive areas with low vulnerability and no presence of indigenous cases, and stratum four, receptive areas, as well as vulnerable places with autochthonous cases. The stratification of Salinas was determined at the third administrative level corresponding to the canton, its risk at levels three and four for malaria transmission (figure 3).

## Discussion

The World Health Organization's guidelines for the elimination of malaria define its reintroduction as the appearance of introduced cases, i.e., the epidemiological connection of cases of local transmission with an imported case in a country or area where the disease had already been eliminated ^6^. Unfavorable sanitary conditions such as the presence of temporary or permanent breeding sites, migratory population flow from endemic areas or countries, and unprepared health systems are triggering conditions for malaria outbreaks.

According to WHO, the detection of three or more cases of malaria per year for three consecutive years is indicative of the re-establishment of malaria transmission ^6^. Sustaining elimination of malaria in areas with high receptivity and vulnerability will require effective strategies to prevent the re-establishment of local transmission ^17^. For this reason, strengthening health services for early diagnosis and timely treatment were the first immediate activities carried out in this canton. Moreover, rapid diagnostic tests were provided to all health care facilities along with specialized training for health personnel and the strengthening of the microscopy diagnostic network.

Due to this outbreak, the city must keep active and reactive surveillance systems activated and, if there is epidemiological silence, carry out proactive searches with an emphasis on the localities where the outbreak occurred. Surveillance and action systems will allow improving decision-making for disease control and elimination. Local community health committees play an important role, especially in attracting migrants from malaria-endemic countries or areas. In this case, the malaria outbreak records were unusual because there was no follow-up of the initial cases.

The first Venezuelan patients reported on April 12 and July 27, 2019, made the local population vulnerable opening the way to their eventual involvement as silent reservoirs of *Plasmodium* spp. making gametocytes available for malaria vectors, which would lead to a stable and continuous transmission ^18^^,^^19^. According to the timeline presented, it is possible that semi-immune patients, such as migrants from malaria-endemic countries, had asymptomatic parasitemia due to long and continuous exposure to parasites in their countries of origin and this took them to seek medical attention when it was already late ^20^.

The majority of patients (57.14%) were men but there were no statistical differences between sexes, which may be explained by the occupation profile of patients since the majority were students (33.3%) and unemployed (33.3%). This would also indicate that contagion was autochthonous in those patients that carried out their activities in the locality, did not mobilize outside the province, and were constantly exposed to mosquito breeding sites. Keeping standing water next to homes for 3 to 5 days has been reported to increase the risk of malaria infection, as well as human activities such as digging the soil and building roads, as this creates temporary pools that provide active breeding sites for mosquitoes and facilitates transmission ^21^^,^^22^.

The most representative age group was 20 to 49 years (n=9; 47.62%), however, three cases were recorded in the 0 to 4 years age group (n=3; 14.29%). The presence of infection in infants and children under 5 implies malaria indigenous transmission of malaria ^23^. The prevalence pattern reflects the state of immunity against malaria as the result of several repeated infections. As no cases had been registered in this town for 6 years, people did not have significant immunity ^23^.

Malaria transmission is determined mainly by human behavior and the existence of the parasite, as well as social factors, housing conditions, population occupation, treatment-seeking behavior, and health facilities. Importantly, a high proportion of the urban population at any age is at risk of malaria due to the absence of acquired immunity ^24^.

One of the first cases recorded in Salinas was a Venezuelan patient who entered the canton two months before presenting symptoms of malaria but reported an epidemiological history in his country. A similar situation was reported in 2018 in El Oro province, where five Venezuelan migrants with no acute symptoms were associated with the malaria outbreak in that province ^25^.

The movement of people, international travel, and migration have been associated with the spread of various arboviruses among local populations ^26^^,^^27^. Due to the socio-economic and humanitarian conditions in Venezuela, since 2014 migration has increased with an estimated two million people moving to neighboring nations such as Colombia, Ecuador, Chile, Brazil, Argentina, and Peru ^28^, countries where, in recent years, an increase in malaria cases has been reported. In 2017 and 2018, Colombia registered 2,048 new *P. vivax* malaria cases imported from Venezuela ^29^.

Malaria in migrant patients has been characterized by mild symptoms with low levels of parasites, less time for *Plasmodium* spp. elimination after treatment, and shorter duration of fever in travelers. Additionally, *P. falciparum* malaria in migrants with symptoms occurred in the first three months after the arrival at endemic countries and the possibility of asymptomatic patients has been contemplated ^30^^,^^31^. In Salinas, the arrival of migrant populations, the suitable climatic factors, and the presence of malaria vectors possibly contributed to the creation of local transmission and the reintroduction of the disease. However, it is not possible to define exactly whether migrants were exposed to malaria in Venezuela or while "in transit" but they represent a highly vulnerable group ^25^.

*Anopheles albimanus* has been identified as the main vector involved in malaria transmission in South America, Central America, and the Caribbean ^32^, especially in places with higher salinity exposed to direct light such as low mangrove areas or near moisture drainage ^33^. In Ecuador, *An. albimanus* is distributed throughout the coastal region below 500 masl including Santa Elena province ^34^.

In this study, the main breeding sites were found in estuaries, wells, and water channels with high larval densities of *An. albimanus.* Those in wells were the second with the highest larval density (28%). The entomological research determined that this type of breeding site exposed to climatic conditions had been abandoned. Other reports have described the presence of *An. albimanus* larvae in Esmeraldas, Manabi, Los Rios, El Oro, and Guayas provinces associated with breeding sites in road ditches, rice paddies, swamps, ponds, river edges, human dwellings, cattle pastures, and plantations ^35^.

In Colombian towns, wells are a common source of water for domestic use in rural areas and are classified as suitable breeding sites for immature mosquitoes and a refuge for *Anopheles, Aedes,* and *Culex* species during the dry season ^36^. It is important to emphasize that factors as the change in land use and the construction of roads and channels are a direct source of malaria vectors breeding sites and, therefore, the proliferation of infectious diseases ^37^. The identification of potential breeding sites in outbreak areas is a key factor for risk assessment and the establishment of more effective control measures in immature vector populations ^38^.

After the interventions, the technical team of the Ministry of Public Health and the National Institute for Public Health Research conducted a situation analysis and concluded that the initial imported cases of malaria in a city with reduced surveillance and low perception of the disease among the local medical personnel, as well as insufficient access to diagnosis in a single health establishment and limited hours, the unavailability of drugs, the lack of knowledge on case-finding and control activities, created the ideal environment for the reestablishment of the disease in an area previously declared as malaria-free.

As part of the activities, the strategic lines of diagnosis, treatment, investigation, and response were strengthened and a stock of medicines was provided both for the parasitic species involved in the outbreak and for *P. falciparum* and five laboratory workers were trained for malaria diagnosis microscopic analysis and provided with rapid diagnostic tests. The epidemiology and vector control team was trained in effective research activities and integrated vector management based on risk stratification. On the other hand, all health facilities in the city were instructed to screen febrile cases for malaria, proactive search activities were planned if no cases are detected in health facilities, as well as routine integrated vector management activities and evaluation of mosquito-net acceptance, with the purpose to align the city to the malaria elimination strategy.

In conclusion, after six years of absence of malaria cases, we present the first report of autochthonous cases of *P. vivax* in the locality of Salinas. This malaria outbreak has important implications for the public, economic, therapeutic, and logistical health-related activity in the city and the province in general. In this sense, a more robust surveillance-response system is essential when moving towards the elimination of malaria. Taking into account the economic burden that the active search of malaria cases represents, we consider that a community-based surveillance system for malaria testing among newcomers in neighborhoods might be a more feasible approach for epidemiological control. Furthermore, the surveillance of infectious diseases along migration routes is crucial to prevent the recurrence of malaria, not only in the province of Santa Elena but also in other localities with the same characteristics.
